# Special Plant Species Determines Diet Breadth of Phytophagous Insects: A Study on Host Plant Expansion of the Host-Specialized *Aphis gossypii* Glover

**DOI:** 10.1371/journal.pone.0060832

**Published:** 2013-04-08

**Authors:** Wei Wu, Xin Li Liang, Hai Yang Zhao, Ting Ting Xu, Xiang Dong Liu

**Affiliations:** Department of Entomology, Nanjing Agricultural University, Nanjing, People’s Republic of China; Centro de Investigación y de Estudios Avanzados, Mexico

## Abstract

Host specialization is a ubiquitous character of phytophagous insects. The polyphagous population is usually composed of some subpopulations that can use only a few closely related plants. Cotton-melon aphids, *Aphis gossypii* Glover exhibited strong host specialization, and the cotton- and cucurbits-specialized biotypes had been clearly identified. However, the experimental work that addressed the roles of plant species in determining diet breadth of phytophagous insects is rare. In the present study, we took the artificial host transfer method to assess the role of two special plants, zucchini *Cucurbita zucchini* L. and cowpea *Vigna unguiculata* (Linn.) Walp, in regulating diet breadth of cotton- and cucurbits-specialized *A. gossypii* collected from cotton and cucumber fields and reared separately on the native host plant for ten years. The results showed that the cotton-specialized aphids did not directly use cucumber whereas the cucurbits-specialized did not use cotton regardless of the coexistence or separation of cotton and cucumber plants. Neither of the cotton- and cucurbits-specialized aphids could use capsicum *Capsicum annuum*, eggplant *Solanum melongena*http://en.wikipedia.org/wiki/Carolus_Linnaeus, tomato *Solanum lycopersicum*, maize *Zea mays*http://en.wikipedia.org/wiki/Carl_Linnaeus, and radish *Raphanus sativus,* however, both of them could use zucchini and cowpea. Moreover, the feeding experience on zucchini led the cotton-specialized aphids to use cucumber well and finally to be transformed into the cucurbits-specialized biotype. The short-term feeding experience on cowpea resulted in the diet breadth expansion of the cucurbits-specialized aphids to use cotton. On the other hand, the diet breadth expansion of the cucurbits- and cotton-specialized aphids was only realized by different species of plant. It concluded that the special host plant did induce the conversion of feeding habits in the cotton- and cucurbits-specialized aphids, and consequently broke the host specialization. The plant species is an underlying factor to determine the diet breadth of phytophagous insects.

## Introduction

Evolutionary direction of feeding habit is various in phytophagous insects. Some species evolve toward polyphagy, and some toward oligophagy or monophagy. Moreover, in polyphagous population, not all the individuals can use all the host plants in their host plant range, and the population is usually composed of several subpopulations which only use a subset of plants in its host range, such as a family or genus plants [1]–[7]. The specialization in habitat or resource use of polyphagous insects might be a common evolutionary trend [8],[9]. Nosile studied the evolution direction of resource use from phylogenies of 15 groups of phytophagous insects including walking sticks, butterflies, beetles, treehoppers and aphids, and found that the forward transition rate from generalization to specialization was significantly higher than the reverse transition rate from specialization to generalization, but specialization did not always represent an evolution dead-end [10]. The study on the nymphalid butterfly tribe Nymphalini showed that there was no directionality in host range evolution toward increased specialization, and changes of host range exhibited a very dynamic pattern [11]. Therefore, specialization of insects might be a phenotype appearing in a specific habitat or at the particular time. Although the hypothesis that specialization does not restrict the further evolution of feeding habits had been proposed, the directional experimental data to support the hypothesis are still rare.

Cotton-melon aphid *Aphis gossypii* Glover is a fine life system to study the evolution of feeding habit because of the short life history, various reproductive modes, and wide host plant range. The previous studies illustrated that cotton-melon aphids had formed the classical host races on some specific host plants within their almost 300 species of recorded host plants [12]–[18]. The fitness of the cotton- and cucurbits-specialized host races on some secondary host plants, such as cotton, cucurbits and chrysanthemum was significantly different, and the cotton- and cucurbits-specialized aphids could not survive if their host plants were alternated [12],[15],[18]. Host plants played an important role in the genetic structure of *A. gossypii* population [19]–[22]. The genotypes of *A. gossypii* on cotton and cucurbits were distinct based on the data from eight microsatellite loci, and the aphids feeding on cucurbits belonged to the *C* genotype whereas the aphids on cultivated malvaceous host plants (cotton, okra, rosella) belonged to the *Burk* and *Ivo* genotypes, although the genetic diversity of *A. gossypii* collected from different locations and host plants was very low [21],[22]. On the other hand, *A. gossypii* reproduced by parthenogenesis mode most of the year. The reproduction mode of *A. gossypii* was exclusive clonal in Europe, and it was mostly asexual with only once sexual in China and some other cool areas [14],[23],[24]. The parthenogenesis on a specific host plant during the annual cycle facilitated the formation and maintenance of host specialization [19],[25] [26]. The performances in host use, reproduction mode and genotype showed that the cotton- and cucurbits-specialized aphids had greater ecological and genetic divergence. However, it is unknown whether the host specialization of cotton-melon aphids can be broken and realize the conversion from specialization to generalization.

The specialization in host use would be one of the scenarios in the evolutionary process of feeding habits in phytophagous insects, because the evolution of host plant use was a highly dynamic process [10]. Host specialization has been thought to be related with the plant chemistry and natural enemies [2],[5]–[7] [27],[28]. The natural enemies drove the host shift and feeding specialization of *Papilio machaon* [5],[29]. The availability of host plants in habitat played an important role in determining the host specialization and diversity in polyphagous insects [9] [30]–[33]. The mites *Tetranychus urticae* collected from cotton improved their fitness on cucumber when they were reared five generations on cucumber, but the fitness on zucchini was no significant difference between the mites reared on cotton and cucumber [34]. However, the experimental studies on host plant induced the fitness variation and diet breadth expansion of host specialized aphids were very few. The present study aimed to find some special host plants which could be used by both the cotton- and cucurbits-specialized aphids, and then to examine whether these special host plants could break the host specialization of *A. gossypii* and realize the conversion of feeding habit from specialization to generalization. The study will attain the direct experimental data to confirm that the special host plant plays an important role in determining the diet breadth of phytophagous insects.

## Materials and Methods

### Ethics Statement

The cotton-melon aphids used here were collected from the Experimental Station of Nanjing Agricultural University. No specific permits were required for the described field studies. We confirm that the location is not privately-owned or protected in any way, and the field studies did not involve endangered or protected species.

### Aphids

Two populations of cotton-melon aphid were collected from cotton *Gossypium hirsutum* and cucumber *Cucumis sativus* fields at the Experimental Station of Nanjing Agricultural University in July 2001, in Nanjing, China, and reared separately on the seedlings of cotton and cucumber by parthenogenesis for ten years in growth chambers (24±1°C, L:D = 14∶10, RH = 70−80%). All aphids were confirmed in terms of their taxonomy by examination of morphological characters and the mtDNA COI gene sequences as *A. gossypii*. The two populations exhibited a strong host fidelity to cotton or cucumber, and they did not survive and establish population on the alternative host plant [18]. Therefore, we classified them into two host specialized biotypes, the cotton-specialized biotype (CO) and cucurbits-specialized biotype (CU). The cultivars of cotton and cucumber used to culture aphids in this study were Nannong 99-8 and Lufeng, respectively.

### Detection of Host Plant Range of the CO and CU

Nine species of crops: cotton, cucumber, zucchini, cowpea, capsicum, maize, eggplant, tomato, and radish which were the crops widely cultivated in Nanjing, were examined separately as the host plant of the CO and CU. In the test experiments, 15–20 adults of the CO or CU were transferred onto the seedlings of each of these plants, and then the adults and their offspring were surveyed every day or at two days interval until the aphids multiplied more than five times or decreased to zero. Here, the growth rate (*R*) of aphids on the tested plant was computed by *R* = *N_t_*/*N_0_*, where *N_t_* was the number of aphids at day *t* after transfer, and *N_0_* was the number of the aphid adults transferred. If the *R* value of aphids on a plant was always more than one at every day, the aphids would well use the plant and flourish, otherwise, the population would use hardly and perish. Therefore, in the present study, we evaluated a plant as a host for the CO or CU by a simple standard that the growth rate of aphids became more than five-fold within 20 days after being transferred onto the plant. The cucumber and cotton were examined ten times for the CO and CU, and the other plants were 30 times.

In order to check whether the coexistence of cotton and cucumber would alter the host use of the CO and CU population, a pair of host plants, one cotton and one cucumber seedlings with five leaves were covered together by a transparent plastic chamber, and 20 adults of the CO or CU were transferred onto the plants (10 aphids on the cotton and the other 10 aphids on the cucumber). In the first five days after transplantation, the aphids on the cotton and cucumber plants were surveyed every day, and then surveyed at two-day interval until all the aphids died. During the experiment, the fresh native host plant for the tested aphids was not added or watered any more when the aphids reached a peak, which would result in the death of the native plant in a chamber and all the aphids would consequently transfer onto the alternative host. But the alternative host plant was added normally when it became poor. For the CO and CU population, four repetitions of transplantation were carried out. If the CO and CU did not survive on the alternative host plant after the native host plant died, we evaluated that the aphids did not directly use the alternative host.

### Experimental Design of Feeding Experience for CO and CU

We found that both the CO and CU could use zucchini and cowpea in the above-mentioned experiments. But it was unknown whether the host plant range of the CO and CU would be altered by the feeding experience on zucchini or cowpea. Therefore, the experiments on feeding experience were designed here. The CO or CU adults were transferred onto zucchini and reared for different generations. Then, 20 adults from the zero, third, fifth, seventh, or seventeenth generation on zucchini were transferred back onto a cotton and a cucumber seedling, and the growth rates (*R*) of population were measured every two days until all the aphids died or the growth rate exceeded fivefold. During the experiment, the host plant, cotton or cucumber was replaced by a fresh one when it became poor. Each of the transfer was repeated 10 times. If the *R* values of the CO on cucumber or the CU on cotton were always more than one, we considered that the CO and CU gained the ability to use the alternative host via the feeding experience on zucchini and realized the expansion of the diet breadth.

Feeding experiences of the CO and CU population on cowpea were also studied for five successive generations (G1–G5) using the same method mentioned above. Fifteen adults of the CO or CU from each generation reared on cowpea were transferred onto cotton and cucumber, and the growth rates of the transferred aphids were recorded every two days. Each of transfers was repeated three times for the CO and CU. Here we only checked the feeding habit of aphids reared one to five generations on cowpea, because five consecutive generations experience on zucchini would alter the feeding habit of aphids according to the result from the above-mentioned experiment.

### Statistical Analysis

The growth rates of the CO and CU aphid population reared different generations on zucchini or cowpea host plant were analyzed by a one-way analysis of variance (ANOVA). Differences of the mean growth rates among different host transfers at a specific date (usually at 8 or 10 days after transfer) were compared by the Duncan’s multiple-range tests at P<0.05. The difference of growth rate of the CO or CU at a specific time between two kinds of transplantations was compared by the student’s t test. All the statistical analyses were performed by software JMP 9.0 (SAS Institute).

## Results

### Host Specialization of Cotton-melon Aphids

**Figure 1:**
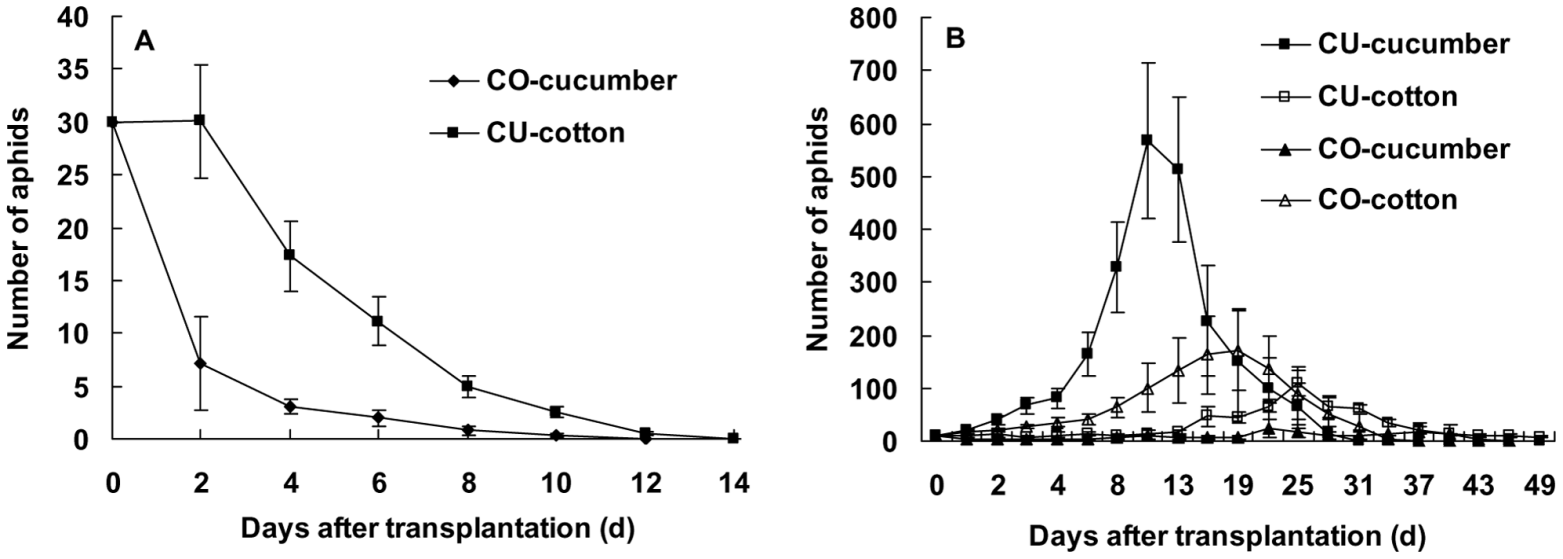
Population dynamics of the cotton-specialized (CO) and cucurbits-specialized (CU) aphids on cucumber and cotton under a non-selective (A), and a selective (B) condition. Bars are means±1SE.

Both the cotton-specialized aphids (CO) and cucurbits-specialized aphids (CU) did not use these plants: capsicum, maize, eggplant, tomato, and radish, on which they only survived two to six days. The CO could not use cucumber whereas the CU could not use cotton. Under a non-selective condition, the CO population survived only 12 days on the seedlings of cucumber, and the CU population survived only 14 days on cotton (Figure 1A). When the cotton and cucumber host plants coexisted, the CO or CU population would settle on both the host plants, but as the native host plant withered away, the aphid population gradually died away too (Figure 1B). Both the CO and CU could not directly establish population on the alternative host plant.

### Intermediate Host Plants of the CO and CU

**Figure 2:**
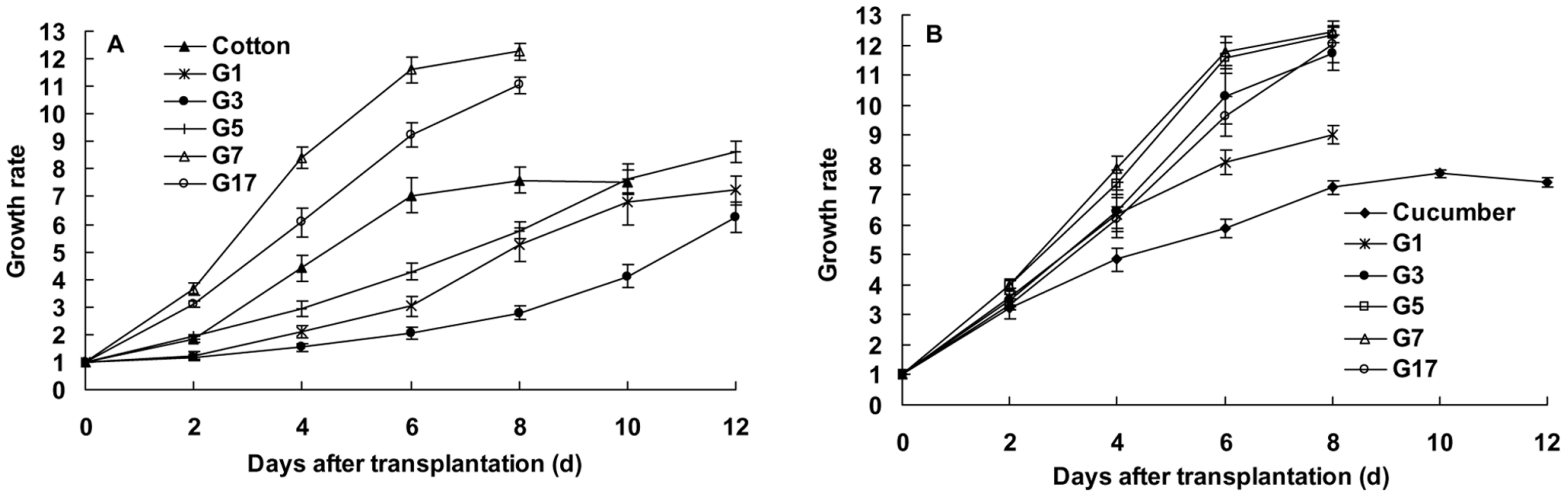
Growth rates of the cotton-specialized aphids (A) and cucurbits-specialized aphids (B) on zucchini for different generations (G1–G17). Bars are means±1SE.

The CO population developed faster on zucchini, and the growth rate of population became significantly higher at the seven and 17 generations on zucchini than that on cotton (at the 8th day after transplantation: F_5, 54_ = 83.177, p<0.0001, Figure 2A). Moreover, the CU population performed better on zucchini rather than on the native host cucumber, and the growth rates were significantly higher on zucchini than that on cucumber (at 8th day after transplantation: F_5, 54_ = 27.548, p<0.0001, Figure 2B).

**Figure 3:**
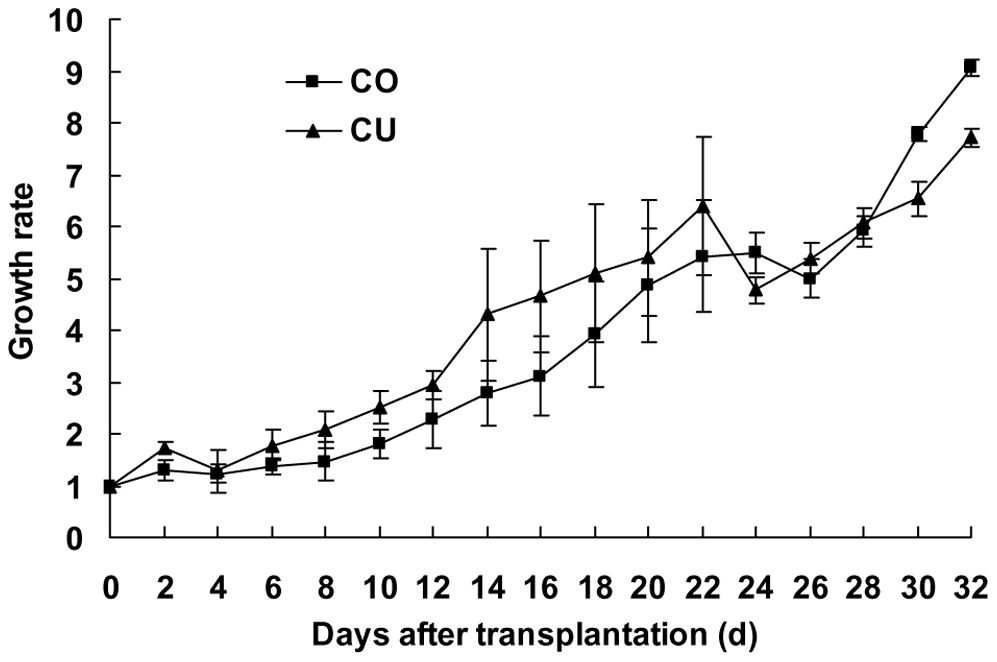
Growth rates of the cotton-specialized aphids (CO) and cucurbits-specialized aphids (CU) on cowpea. Bars are means±1SE.

The CO and CU population increased stably on cowpea, and the growth rates at 32 days after transplantation were nine and eightfold for the CO and CU, respectively. The CO and CU population could share the cowpea host plant (Figure 3).

### Intermediate Hosts Alter Host Specialization of the CO and CU

**Figure 4:**
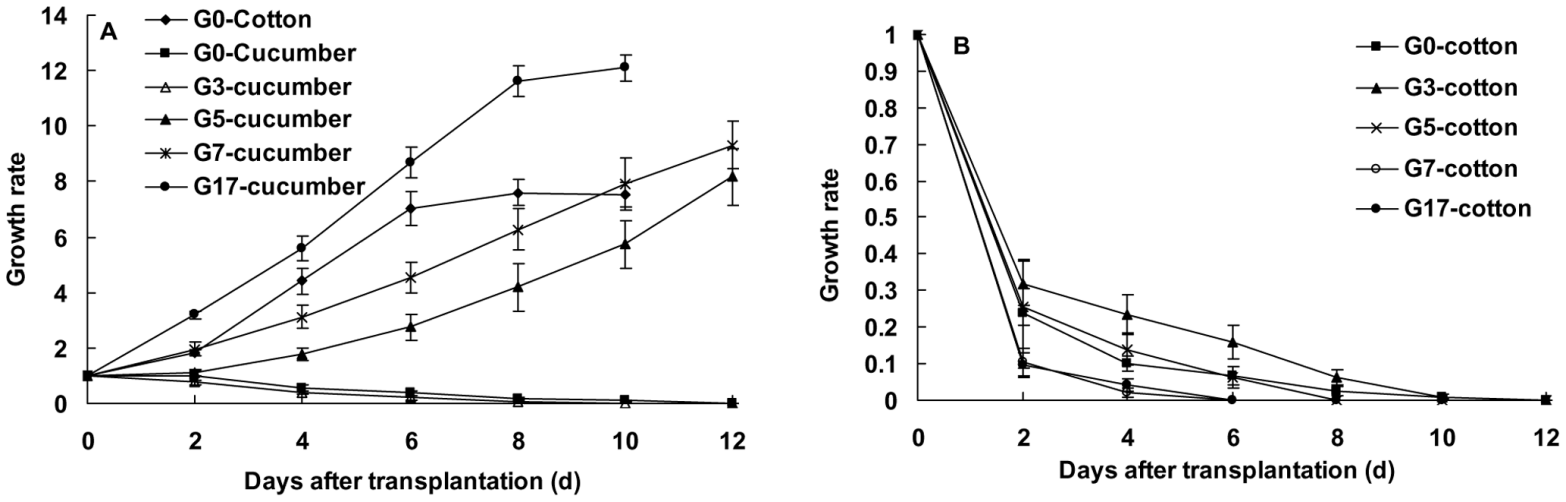
Growth rates of the cotton-specialized aphids on cucumber (A) and the cucurbits-specialized aphids on cotton (B). The transferred aphids were reared for zero, 3, 5, 7, and 17 generations (G0–G17) on zucchini. Bars are means±1SE.

The feeding experience on an intermediate host plant, zucchini altered the feeding habit of the CO to use cucumber, and this kind of alteration only happened after the CO had been reared five or more generations on zucchini. The growth rate of the CO population was less than one and decreased to zero at the eighth day after being transferred onto cucumber when the population had experienced only three generations on zucchini. However, the growth rate increased to six times at the tenth day on cucumber as the population had experienced five generations on zucchini. Moreover, the growth rate of the CO population on cucumber became significantly higher than that on cotton when they had been reared 17 generations on zucchini (at 8th day after transplantation: t = 5.397, *df* = 18, p<0.0001, Figure 4A). Although the CU used zucchini very well, they did not gain the ability from zucchini to use the cotton plant, even if they had been lived on zucchini for three to 17 generations. The CU reared on zucchini for zero to 17 generations could survive only six to ten days on cotton (Figure 4B).

**Figure 5:**
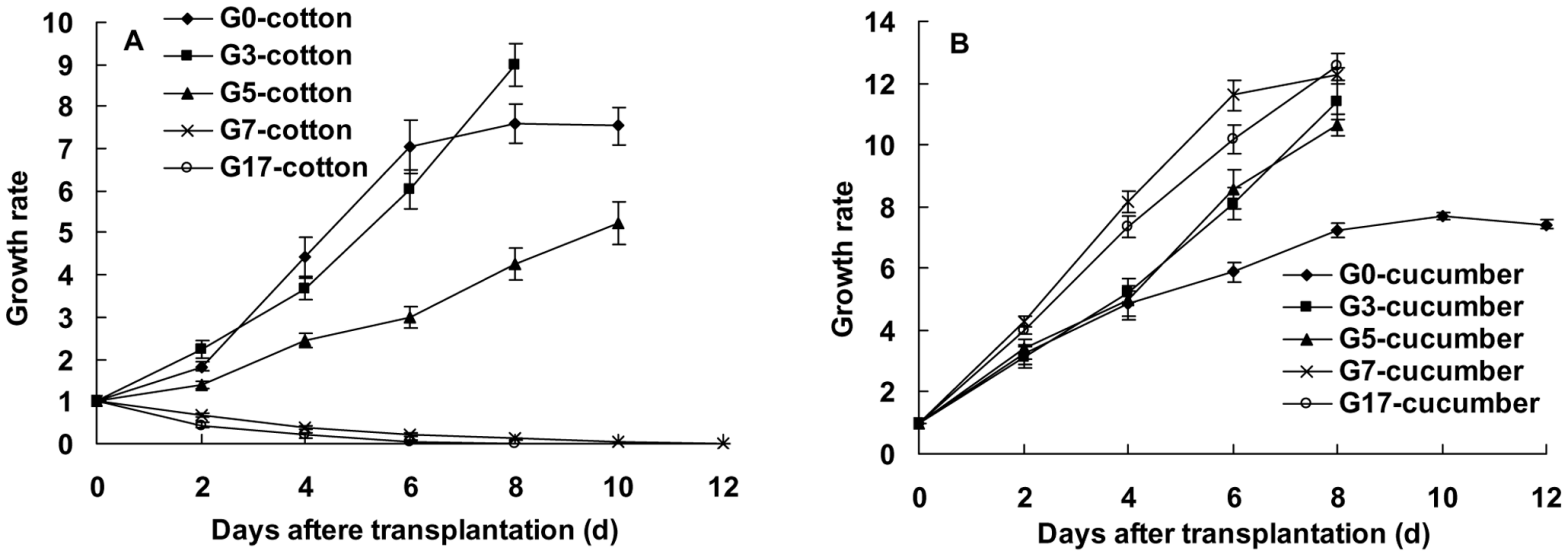
Growth rates of the cotton-specialized aphids on cotton (A) and the cucurbits-specialized aphids on cucumber (B). The transferred aphids were reared for zero, 3, 5, 7, and 17 generations (G0–G17) on zucchini. Bars are means±1SE.

The feeding experience on zucchini led the CO to reduce or lose the ability to use the native host plant, cotton. The CO was still possessed of the ability to use cotton if it lived on zucchini only three or five generations. However, after seven generations or more, the CO did not normally use cotton plant again, and the population size declined gradually (Figure 5A). The result illustrated that the cotton-specialized biotype had successfully converted into the cucurbits-specialized biotype. On the other hand, the CU became more and more suitable for using cucumber when it lived on zucchini for three to 17 generations, and the growth rates of population reared on zucchini for different generations were significantly higher on cucumber than that of the population reared on the native host plant, cucumber (at 8th day after transplantation: F_4, 45_ = 29.097, p<0.0001, Figure 5B).

**Figure 6:**
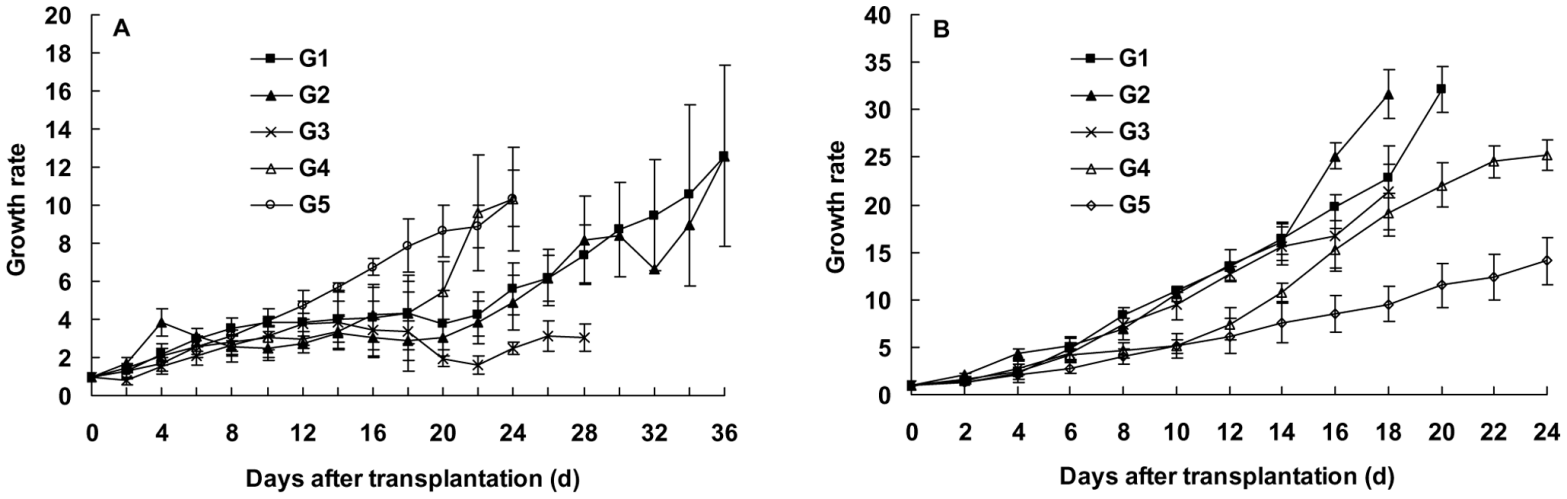
Growth rates of the cucurbits-specialized aphids on cotton (A) and on cucumber (B). The transferred aphids were reared for 1–5 generations (G1–G5) on cowpea. Bars are means±1SE.

The feeding experience on cowpea led the CU to use both the cucumber and cotton plants, although the growth rate of population on cotton was relatively lower than that on cucumber (Figure 6A and B). The feeding experience on a novel host plant cowpea extended the diet breadth of the CU aphids, but it did not wholly alter their preference for the native host plant cucumber. Nevertheless, the growth rate of the CU population on cucumber exhibited a declined trend as the generation of experience on cowpea increased, and the growth rate of the CU population reared five generations on cowpea became significantly lower than that reared one or two generations (at 10 days after transfer: F_4,10_ = 7.988, P = 0.0037, Figure 6B).

**Figure 7:**
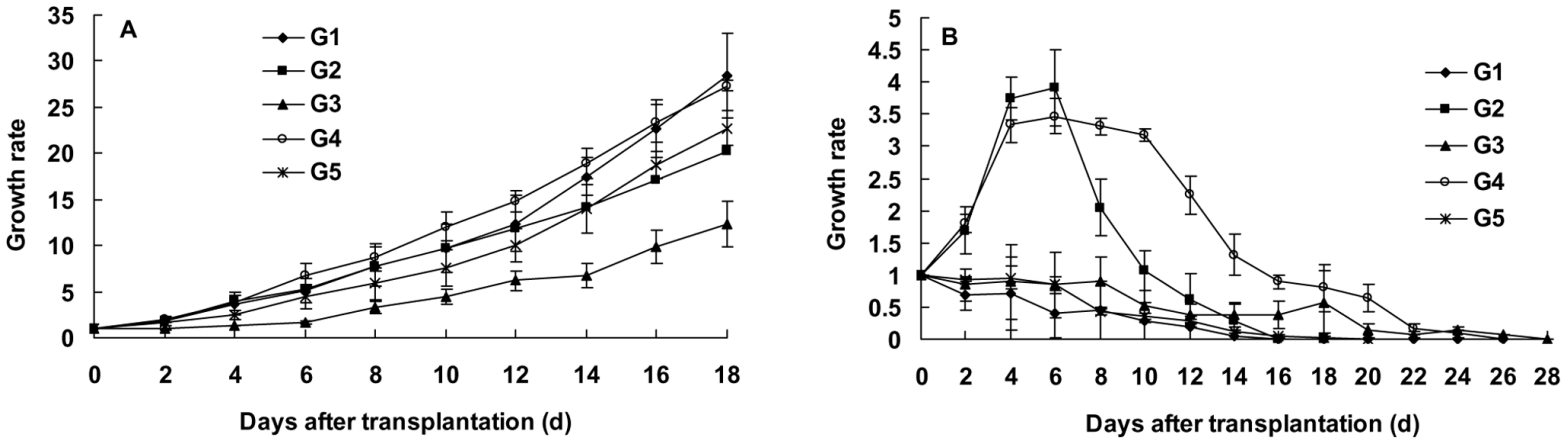
Growth rates of the cotton-specialized aphids on cotton (A) and on cucumber (B). The transferred aphids were reared for 1–5 generations (G1–G5) on cowpea. Bars are means±1SE.

The CO reared on cowpea for five generations did not lose the ability to use cotton, and the population increased steadily (Figure 7A). Moreover, the feeding experience on cowpea did not alter the host preference of the CO for cotton. The CO still could not use the cucumber when they were reared on cowpea for five generations, and their growth rates declined quickly after six days on cucumber till became zero at 16 to 28 days. There were no improved trends of the CO on cucumber when the experienced generation increased on cowpea (Figure 7B).

Overall, the intermediate host zucchini altered the feeding habit of the CO to use cucumber, and the cowpea expanded the diet breadth of the CU to use cotton. The conversion of feeding habit of cotton-melon aphid from specialization to generalization had been realized by the feeding experience on an intermediate host plant.

## Discussion

Our results showed that the cotton-specialized aphids could not directly use cucumber, and the cucurbits-specialized aphids could not use cotton too, but both of them could use zucchini and cowpea. The fact that the two host-specialized biotypes of *A. gossypii* still shared the same host plants illustrated that the specialized aphid was not wholly constrained on a species of host plant. Although the specialists have obvious advantages in predator avoidance and decision of host plants [17],[35] [37], they would be likely to meet the high risk of host lack, especially in an inconstant agroecosystem. Maintaining the ability of the cotton- and cucurbits-specialized aphids to use zucchini and cowpea was undoubtedly favorable for population when the aphids did not encounter their native host plant during the dispersal or migration, because the two plants, zucchini and cowpea usually coexisted synchronously with cotton and cucumber in the summer, in Nanjing. The previous studies also illustrated that both the cotton- and cucurbits-specialized aphids had the ability to use *Hibiscus syriacus*, an overwintering host plant which could act as a refuge plant between two vegetable crop seasons [18],[21]. The refuge plant would act as a transition host for feeding or reproduction of the specialized aphids during the dispersal or migration from an innutrient environment to a better one. Additionally, the different specialized genotypes of *A. gossypii* had been found on *H. syriacus* [21]. The vegetable crops, zucchini and cowpea would act as the refuge plants in the summer for both of the cotton- and cucurbits-specialized aphid *A. gossypii*.

The feeding experience on zucchini and cowpea altered the preference of *A. gossypii* for cotton and cucumber. The cotton-specialized aphids feeding on zucchini could gain the ability to use cucumber, and the cucurbits-specialized aphids feeding on cowpea could gain the ability to use cotton. The zucchini and cowpea were the intermediate hosts to realize the host range expansion or host shift of *A. gossypii*. Host plant chemistry was considered as a factor that best explained the macroevolutoinary patterns of host use in herbivorous insects. Host shifts by phytophagous insects could sometimes be mediated by plant chemical similarity [38]–[41]. The result here that zucchini altered the cotton-specialized aphid’s feeding habit could be explained well by the plant chemical similarity hypothesis, because the host zucchini belongs to the same family Cucurbitaceae as cucumber and contains the cucurbitacins which resulting in feeding and oviposition deterrence and reduced growth of herbivorous consumers [42]–[44]. The ‘melon strain’ of *A. gossypii* adapted the pyrazole compound, a secondary chemistry in melon very well whereas the ‘cotton strain’ did not tolerate the higher doses of pyrazole, although the physiological concentrations in melon phloem sap did not harm aphids [45]. If the ‘cotton strain’ was contiguously selected by melon, it would acclimatize the stress of pyrazole. In the present study, we found that the cotton-specialized aphids lived five generations on zucchini gained the ability to use cucumber, but then lost the ability to use their native host cotton after seven generations. It implied that the zucchini would contain the same chemical inhibitors as the cucumber which suppressed the cotton-specialized aphids to use, but unlike cucumber, the dose of the inhibitors might be insufficient to cause all the cotton-specialized aphids death. Therefore, the cotton-specialized aphids acclimatized themselves to zucchini after living five generations and sequentially could use the cucumber after seven generations. However, the phenomena that the cowpea altered the cucurbits-specialized aphid’s feeding habit to use cotton seem would not be simply explained by the plant chemical similarity hypothesis, because the cowpea, cucumber, and cotton belong to different plant families and contain different secondary compounds. Even so, we presumed that there must be a chemistry mechanism in cowpea to improve the ability of the cucurbits-specialized aphids to overcome the defense system of cotton plant, but the actual mechanism was unclear presently.

However, we did not find an intermediate host plant which could alter both the cotton- and cucurbits-specialized aphid’s feeding habit. The different routes to realize diet expansion or host shift for cotton- and cucurbits-specialized aphids are favorable for maintenance of the specialization of *A. gossypii* in nature. The cotton-specialized aphids reared on zucchini for seven generations had realized the whole conversion from the cotton biotype to cucurbits biotype. However, the cucurbits-specialized aphids could not realize the conversion from the cucurbits biotype to cotton biotype after they were reared on cowpea. The result suggested that the cucurbits-specialized aphids would be derived from the cotton-specialized aphids. The identical sequences of mtDNA COI gene in cotton- and cucurbits-specialized biotypes of *A. gossypii* were also supported this conversion pathway [46]. The formation route of the cucurbits-specialized biotype of *A. gossypii* from cotton-specialized biotype via intermediate host plant zucchini revealed that the variation of plant species in habitat would drive the feeding habit change of aphids. In some other phytophagous insects, the enemy-free space and host geographical similarity also promoted the formation of host specialization [2] [5] [27] [41]. The specialization in different species or genus of phytophageous insects would be driven by different selective pressures. The reversibility of specialization of *A. gossypii* into generalization via a specific host plant found in the present study supplies a direct evidence to confirm the Oscillation Hypothesis that lineages of phytophageous insect could alternate between generalist and specialist phases [47],[48] and the specialization is not a dead-end in the evolution of feeding habit [10]. The special host plant played an important role in driving the change of feeding habit. Of course, the present study only detected the conversion of feeding habit of host specialized *A. gossypii* in laboratory by artificially host transfer method, and the actual case of the specialized aphids in the natural fields still needs to be surveyed in the future. Undoubtedly, the experimental data in the present study were sufficient to confirm that the intermediate host plant plays an important role in determining the diet breadth of melon-cotton aphids.
